# Efficient removal of tetracycline with KOH-activated graphene from aqueous solution

**DOI:** 10.1098/rsos.170731

**Published:** 2017-11-29

**Authors:** Jie Ma, Yiran Sun, Fei Yu

**Affiliations:** 1 State Key Laboratory of Pollution Control and Resource Reuse, College of Environmental Science and Engineering, Tongji University, 1239 Siping Road, Shanghai 200092, People's Republic of China; 2 College of Chemistry and Environmental Engineering, Shanghai Institute of Technology, Shanghai 2001418, People's Republic of China

**Keywords:** tetracycline, KOH-activation, adsorption, graphene, specific surface area

## Abstract

Activated graphene absorbents with high specific surface area (SSA) were prepared by an easy KOH-activated method, and were applied in absorbing antibiotics, such as tetracycline (TC). After activation, many micropores were introduced to graphene oxide sheets, leading to higher SSA and many new oxygen-containing functional groups, which gave KOH-activated graphene excellent adsorption capacity (approx. 532.59 mg g^−1^) of TC. Further study on the adsorption mechanism showed that the Langmuir isotherm model and the pseudo-second-order kinetic model fitted with experiment data. To further understand the adsorption process, the effects of solid–liquid ratio, pH, ionic strength and coexisting ions were also investigated. The results revealed that, compared with pH and ionic strength, solid–liquid ratio and coexisting ions (Cu^2+^, CrO_4_^2−^) had more significant influence over the adsorption performance. The findings provide guidance for application of KOH-activated graphene as a promising alternative adsorbent for antibiotics removal from aqueous solutions.

## 1. Introduction

Tetracycline (TC), as one of the most widely used antibiotics all over the world [1], is extensively used for human therapy and agricultural purposes [2], such as growth promoters [3], due to its great therapeutic values, broad-spectrum, high quality and low cost [4]. It is estimated that 2.3 million kg of tetracycline was used for pigs breeding every year in the late 1990s in the USA [3]. However, TC is poorly absorbed and metabolized [5,6] and has a long environmental half-life [6]. Most TC is discharged into the environment through urine, faeces [3,7], municipal wastewater treatment plants and agricultural run-off [8–11]. Residues of TC even below the threshold levels (46–1300 ng l^−1^ of TC [12]) have serious potential adverse effects [1] on the target organisms, including acute chronic toxicity, endocrine disruption, antibiotic-resistant genes [2,13–15] and so on. Therefore, it is urgent to develop efficient and economical methods and technologies to remove the residues of TC in the environment.

Many physico-chemical methods, such as adsorption [16], photochemical processes and electrochemical processes [17] have been applied in removing TC in the water environment. The main drawbacks of the advanced oxidation are the expensive operating costs and energy consumption [2]. Adsorption is generally regarded as an affordable and feasible method for removing heavy metals in natural streams and waste effluents [18–20]. Since the application of the adsorption method in the removal of TC is low cost, efficient and convenient to use, more and more attention has been drawn to it. Many adsorbents, for instance montmorillonite [21], chitosan particles [22], activated carbon [23], carbon nanotubes [24] and graphene [25,26], have been used to remove TC in water. Among them, carbon nanomaterials, especially graphene, are gaining popularity.

Graphene, a two-dimensional carbon nanomaterial with large theoretical specific surface area (SSA) of approximately 2630 m^2^ g^−1^, has received extensive attention in many fields [27,28], because of its excellent electrical, mechanical and thermal properties [29,30]. Despite being a low-cost precursor for graphene, graphene oxide (GO) contains various oxygen-containing functional groups such as epoxy, hydroxyl and carboxylic groups on the surface [15,31]. The functional groups of GO make it hydrophilic, easily disperse in water and capable of adsorbing environmental contaminants, such as methylene blue (MB) [32], methyl orange (MO) [33], heavy metal [34] and antibiotics [26]. Wang *et al.* [34] studied the adsorption behaviour of reduced graphene oxide (RGO) for Pb(II), Cd(II), Cu(II) and Mn(II), and showed the feasibility of RGO in metal recovery and removal. Li *et al.* [35] and his team applied GO/CA to the removal of MB dyes. The SSA of GO, however, is relatively low due to corrugations and aggregation. The adsorption efficiency is identified to be dependent on SSA, porosity, pore diameter and functional groups of adsorbents [2,36]. And physical activation methods and chemical activation methods have been used to obtain high-efficiency adsorbents [37].

In this study, activated graphene adsorbent with higher SSA was synthesized by the KOH-activation method. The prepared KOH-activated graphene was firstly used as an adsorbent in the aqueous solutions and showed excellent adsorption performance towards TC. The adsorption performance and the effects of solid–liquid ratio, pH, ionic strength and coexisting ions were further studied and analysed, to explore whether KOH-activation is a feasible and effective method to enhance the adsorption performance of GO towards TC. Consequently, KOH-activated graphene was considered as a promising adsorbent to remove antibiotics in aqueous solutions.

## 2. Experimental set-up

### 2.1. Materials

All chemicals used in the work were supplied by the Sinopharm Chemical Reagent Co., Ltd (Shanghai, China). The purity of the chemicals are analytically pure and they were used in the experiments directly without any further treatment and purification. All the solutions used within the whole process of experiments were prepared with DI water.

### 2.2. Activated treatment of graphene oxide

The graphite oxide was prepared by a modified Hummer's method [38–41]. The graphite oxide solution was first exfoliated for 12 h by an ultrasound bath. Then the sonicated solution was centrifuged to remove unexfoliated graphite oxide particles, and the freeze-drying method was conducted to prepare GO powder. A mixture of GO and KOH powder (weight ratio of 1 : 4) was heated to 1023 K for 1 h in a tube furnace under flowing Ar atmosphere, then the mixture was washed with concentrated HCl solution and DI water and finally dried. The KOH-activated GO was marked G-KOH and GO was marked G.

### 2.3. Characterization methods

The morphological characteristics and feature of the G and G-KOH samples were investigated by transmission electron microscopy (TEM, JEOL, Japan). The SSA and pore structures of samples were analysed from the N_2_ adsorption/desorption isotherms at 77 K by the multi-point BET and the Barrett–Joyner–Halenda (BJH) method. To study the phase and crystallinity of samples, the X-ray photoelectron spectroscopy (XPS) was executed by a Kratos Axis Ultra DLD spectrometer with monochromated Al Ka X-rays. The information of functional groups can be obtained from Fourier transform infrared (FT-IR) spectra through a Tensor 27 FT-IR spectrometer. Raman spectroscopy (Jobin Yvon T64000) was used to characterize the detailed structure information of G and G-KOH.

## 3. Batch adsorption experiments

Batch adsorption experiments were carried out in duplicate and the average values were adopted. The maximum deviation for the duplicates was usually less than 5%; 5 mg absorbents and 40 ml TC solution were put in 50 ml glass bottles and processed within an incubator shaker at a frequency of 100 r.p.m. The initial concentration of TC is 0–70 mg l^−1^. Meanwhile, the blank experiments without absorbents were also conducted to confirm that the decrease of TC concentration was because of absorbents instead of any other factors. After the adsorption process, the TC concentration was measured by the UV spectrophotometer at the peak of *λ*_max_ = 356 nm [43]. A calibration curve between absorbance and concentration of TC (0 ∼ 20 mg l^−1^) was constructed according to the Beer–Lambert's Law. For solutions with concentration higher than 20 mg l^−1^, the solutions were first diluted with DI water.

Kinetic studies were performed at a constant temperature of 25°C and 100 r.p.m. with 50 mg l^−1^ initial concentration of TC solutions with different adsorption time. The solid–liquid ratio experiments were conducted in 100 mg l^−1^ TC solutions with varying solid–liquid ratio from 1 : 8 to 1 : 2. The effect of solution pH on TC removal was studied in the range of 3–10 with 100 mg l^−1^ initial concentrations of TC solutions. The initial pH values of all the solutions were adjusted using 0.1 mol l^−1^ HCl or 0.1 mol l^−1^ NaOH solution with desired concentrations. The ionic strength experiments were conducted in 100 mg l^−1^ TC solutions with varying ionic strength range from 0 to 2.25 mmol l^−1^. The effect of coexisting metal ions on TC removal was studied with 10–80 mg l^−1^ initial concentrations of TC solutions and 20 mg l^−1^ initial concentrations of Cu^2+^ and CrO_4_^2−^.

The adsorbed amount of TC on the adsorbents (*q*_e_, mg g^−1^) was calculated as follows:

3.1 $$q_{e}=\text{( }C_{0}-C_{t}\text{) }\times \frac{V}{m},$$

where *C*_0_ and *C*_t_ represent the TC concentrations at the beginning and end of the adsorption process (mg l^−1^), *V* represents the initial solution volume (l) and *m* represents the adsorbent weight (g).

## 4. Results and discussion

### 4.1. Characterization of the adsorbents

**Figure 1.**
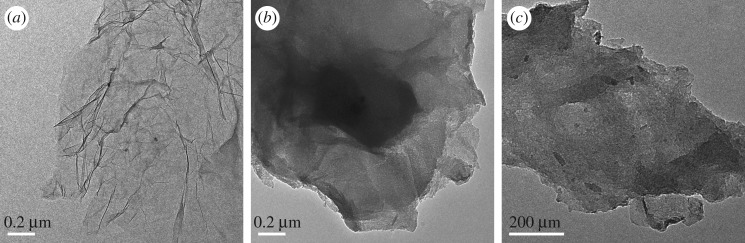
TEM images of G (*a*) and G-KOH (*b*,*c*).

The morphology characteristics and microstructure of the prepared graphene oxide (G) and KOH-activated graphene (G-KOH) were investigated by the TEM. As shown in figure 1*a*, G consists of a few layers graphene and the light-grey films resemble the silk wave [27], which is thin, connected and transparent. Figure 1*b*,*c* indicate KOH-activated GO is inhomogeneous, unconnected and non-transparent, showing that graphene structure has been etched during the activation process, and graphene sheets were divided into much small plats [27].

To further investigate the property of G and G-KOH, the FT-IR, XPS, Raman and N_2_ adsorption/desorption analysis were performed and the results are presented in table 1. Some oxygen-containing functional groups, such as C–O, C=O and –OH, were identified before and after activation, which may act as anchoring sites for TC. As shown in the XPS survey scan results in electronic supplementary material, figure S1, the oxygen atomic content of G-KOH (9.14%) is higher than that of G (6.61%), indicating that the new oxygen-containing functional groups are introduced into the G-KOH. To further analyse the functional groups on the surface, the G and G-KOH samples were characterized by FT-IR (electronic supplementary material, figure S2). The intensity of peaks at 1631 cm^−1^ (C=O stretching) and 3435 cm^−1^ (–OH stretching) increased, confirming that new oxygen-containing functional groups, such as carboxyl and hydroxy groups, were introduced into the G-KOH surface. As for the Raman spectrum, the G peak appearing at 1600 cm^−1^ is related to E_2 g_ graphite mode, which illustrates the graphitization degree of the graphene. The D peak at around 1329 cm^−1^ is related to a defect-related vibration mode due to disordered carbon, edge defects or other defects, such as sp^3^-bonded carbon and topological defects. The intensity ratio of the D and G peak (*I*_D_/*I*_G_) was used to estimate the quantity of defect in the graphene. As shown in electronic supplementary material, figure S3, the *I*_D_/*I*_G_ of G-KOH (1.14) is lower than that of G (1.44), revealing that there are more defects on the G-KOH samples. More structure properties are obtained from SSA and pore volume information. As shown in electronic supplementary material, figure S4, the SSA of G-KOH increases more markedly than G (approx. 3.7 times) and the average pore size decreased approximately from 3.946 to 1.658 nm. The above observations can be ascribed to the mechanism that the graphene sheets were etched and many micropores were introduced at the same time. And the excellent structural properties of KOH-activated graphene, such as higher SSA, more functional groups, create favourable conditions for TC adsorption.

**Table 1.** Physical property of G and G-KOH. C%, the carbon atomic content; O%, the oxygen atomic content; *I*_D_, the intensity of D-Raman peak; *I*_G_, the intensity of G-Raman peak.

samples	C%	O%	*I*_D_/*I*_G_
G	93.39	6.61	1.14
G-KOH	90.86	9.14	1.44

## 5. Adsorption isotherms

**Figure 2.**
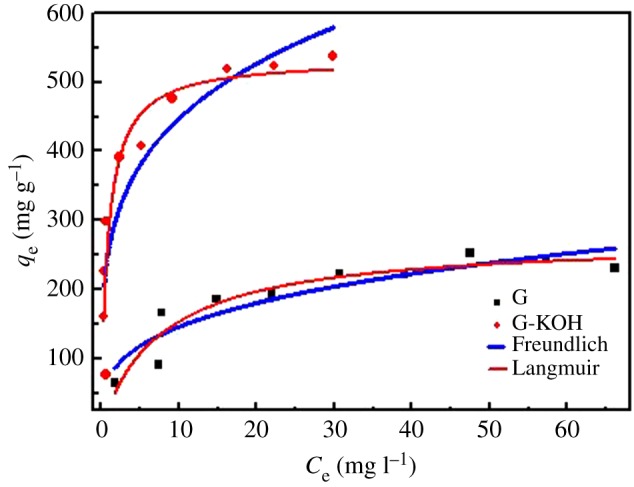
Equilibrium adsorption isotherms of TC on G and G-KOH.

The adsorption isotherms of TC on G and G-KOH were constructed to facilitate better understanding of the adsorption process. In figure 2, *C*_e_ (mg l^−1^) represents equilibrium concentrations, namely the final solution concentrations after the saturated adsorption. The equilibrium adsorption amount (*q*_e_, mg g^−1^) stands for the variation of initial (*C*_0_) and equilibrium concentration (*C*_e_) caused by adsorbent of per unit weight [15]. The data of equilibrium adsorption isotherms was fitted by the Langmuir and Freundlich models. The fitting parameters of the adopted fitting models are listed in table 2. According to the results, the correlation coefficients of the Langmuir equation for G and G-KOH (0.89, 0.84, respectively) are higher than that of the Freundlich equation (0.84, 0.81, respectively). Consequently, the Langmuir isotherm fits the adsorption data better, which is in accord with reported works [12]. The maximum monolayer adsorption capacity calculated by the Langmuir equation was 272.70 mg g^−1^ for TC on G and 532.59 mg g^−1^ on G-KOH, indicating that the adsorption capacity for TC was improved approximately two times after KOH-activation treatment. The improvement of G-KOH might be partly due to the improvement of SSA of G-KOH (512.65 m^2^ g^−1^) compared with that of G (138.2 m^2^ g^−1^), which provides more adsorption sites. From the FT-IR and XPS analysis results, it is found that new oxygen-containing functional groups are introduced into graphene after activation treatment, thereby making the G-KOH more disperse in water and providing more adsorption sites. The enhancement of hydrophilicity increases the contacted area between TC and G-KOH, meanwhile more adsorption sites facilitate the TC adsorption. Therefore, the higher adsorption capacity for TC on G-KOH might also be attributed to the increased oxygen atomic content of G-KOH. To evaluate the adsorption performance of G-KOH, activated carbon [44], NaOH-activated carbon [45] and some other materials used to adsorb TC are listed in table 3. Given that the different properties of adsorbents were used and the experiment was performed under different conditions, it is difficult to estimate the performance of absorbents precisely. Nevertheless, the comparison provides the qualitative information that G-KOH turned out to be a promising effective adsorbent to remove antibiotics in aqueous solutions.

**Table 2.** Langmuir and Freundlich isotherm parameters for TC of G and G-KOH.

	Langmuir model			Freundlich model		
absorbent	*K*_L_ (l mg^−1^)	*q*_m_ (mg g^−1^)	*R*^2^	*K*_F_	1/*n*	*R*^2^
G	0.13	272.70	0.89	72.70	0.30	0.84
G-KOH	1.13	532.59	0.84	260.35	0.24	0.81

**Table 3.** Maximum adsorption capacity (*q*_m_) of different adsorbents for TC.

adsorbent	*q*_m_ (mg g^−1^)	initial concentration (mg l^−1^)	pH	refs.
graphene oxide-MPs	39.1	60	3–10	[46]
graphene oxide	313	266	3–11	[15]
graphene oxide	323	180	6–7	[43]
activated carbon	308.33	80	1.5–8.5	[44]
NaOH-activated carbon	455.83	300	3–10	[45]
G	272.7	30	3–10	this work
G-KOH	532.59	70	3–10	this work

The separation factor *R*_L_, a dimensionless constant of the Langmuir isotherms, was used in evaluating whether the adsorption process is favourable. *R*_L_ was defined by Weber & Chakkravorti [42] as

5.1 $$R_{L}=\frac{\text{1}}{\text{1}+K_{L}C_{0}}.$$

As shown in electronic supplementary material, table S1, the *R*_L_ values of G and G-KOH (0.10 and 0.029, respectively) were found between 0 and 1, suggesting that the adsorption process was quite favourable, and G-KOH had a better adsorption capacity for TC [43].

## 6. Adsorption kinetics

**Figure 3.**
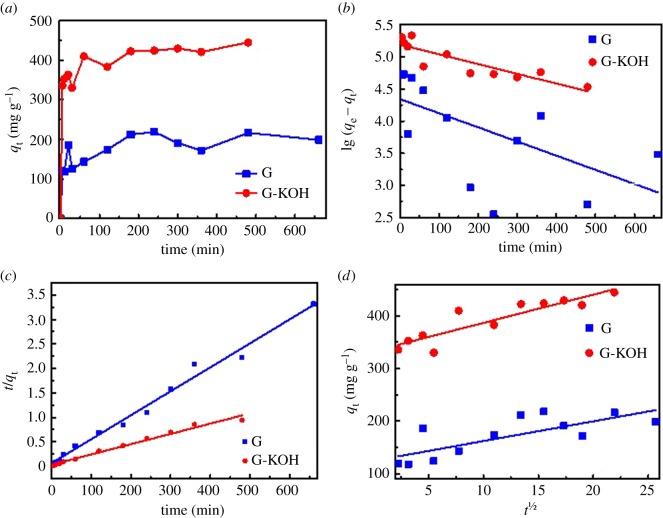
(*a*) Kinetic curves, (*b*) pseudo-first-order model, (*c*) pseudo-second-order model and (*d*) Weber–Morris model.

The adsorption kinetics were also investigated and are shown in figure 3. It was found that the adsorption capacities increased rapidly at the initial period (approx. 15 min). Then the growth of adsorption capacities slowed down within a period of time (approx. from 15 to 100 min). And finally equilibrium was almost achieved in 200 min. To evaluate the kinetics of adsorption of TC on absorbents, the pseudo-first-order, pseudo-second-order (PSO) and intra-particle diffusion models were used to simulate the experimental data. The kinetic parameters and the correlation coefficients (*R*^2^) of three models are given in electronic supplementary material, table S4. In terms of the correlation coefficients, only the results of the PSO model are consistent with the kinetic data of G and G-KOH at 99 and 98 confidence level, respectively. The assumption adopted in the PSO model states that the rate-limiting step involves chemisorption [47], which has been widely used in the analyses of the adsorption of contaminants from solutions [48]. It can be found from electronic supplementary material, table S4 that the calculated *q*_e_ values (*q*_e,cal_) of the PSO model on G and G-KOH are 250 and 500 mg g^−1^, respectively, which are fairly close to the experimental data (*q*_e,exp_). Thus, the PSO model is suitable for fitting the adsorption process of TC on G and G-KOH.

If we neglect the movement of adsorbate from the bulk liquid to the liquid film surrounding the adsorbent, the adsorption process from liquid to porous solids can be divided into three stages [27]: film diffusion, intra-particle diffusion and adsorption onto the active sites. The Weber–Morris equation was used to fit the experimental data, which is further adopted in analysing the rate-controlling step of removal for TC on G and G-KOH samples. As shown in figure 3*d*, the fitting line does not pass through the origin, illustrating that the intra-particle diffusion is not the only controlling step [49]. The large intercept of linear portion of the plots indicates that the external mass transfer has a significant effect on the rate-controlling step.

## 7. Effect of solid–liquid ratio

**Figure 4.**
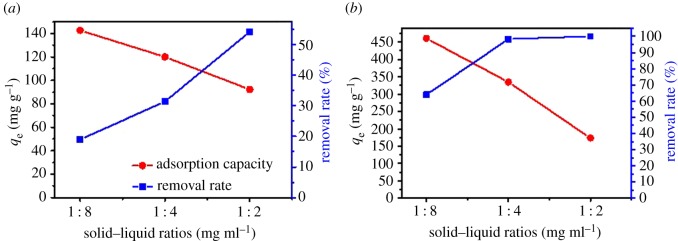
The effect of solid–liquid ratio on adsorption performance on G (*a*) and G-KOH (*b*).

The effect of the solid–liquid ratio on the adsorption performance of TC on GO before and after activation treatment is shown in figure 4. The solid–liquid ratio studied in the experiment varied from 1 : 8 (5 mg absorbent, 40 ml TC solution, 100 mg l^−1^) to 1 : 4 (5 mg absorbent, 20 ml TC solution, 100 mg l^−1^) and 1 : 2 (5 mg absorbent, 10 ml TC solution, 100 mg l^−1^). The results indicate that the adsorption capacity of TC declined greatly with the decrease of the ratio from 1 : 8 to 1 : 2 on G (from 142.68 to 92.26 mg g^−1^) and G-KOH (460.95 to 173.3 mg g^−1^) absorbents. The removal rates of TC on G and G-KOH absorbents both increased with the solid–liquid ratio (from 18.99 to 54.21% and from 63.87 to 99.98%, respectively). In different solid–liquid ratio experiments, the mass of absorbent and the concentration of solution is fixed, on the other hand, the volume of solution decreased, namely active adsorption sites of TC are fixed but the mass of pollutants (TC) decreased with the solid–liquid ratio [50,51]. It is found that the adsorption capacity decreased with increasing solid–liquid ratio, which might be attributed to the split in the flux or the TC concentration gradient between the solution and the surface of the absorbents [51]. As a result, the adsorption capacity decreased with the increase of solid–liquid ratio.

## 8. Effect of pH and ionic strength

**Figure 5.**
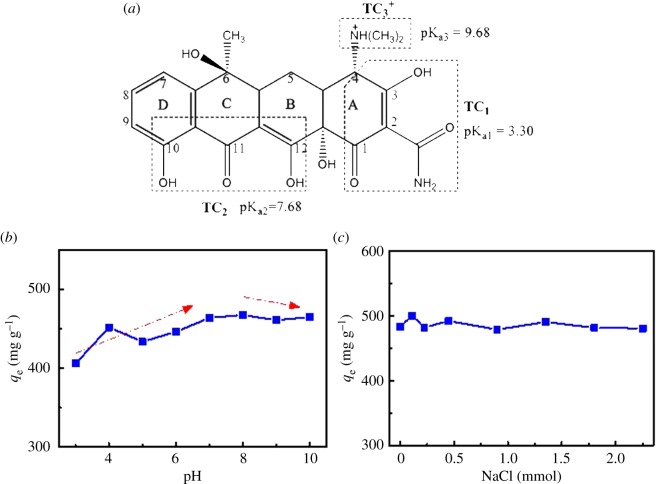
Structure of tetracycline (*a*), effect of pH values (*b*) and ionic strength (*c*) on adsorption of TC on G-KOH.

As shown in figure 5*a*, the charges of tetracycline vary with the pH value of the solution. TC has three different acid dissociation constants (p*K*_a_ = 3.3, 7.7 and 9.7), which appears in the form of cation (TCH_3_^+^), zwitterion (TCH_2_^0^) and anion (TCH^−^ or TC^2−^) under acidic, moderately acidic to neutral and alkaline conditions [52], respectively. The effects of the pH value on adsorption of G-KOH are presented in figure 5*a*. It can be found that the adsorption of TC on G-KOH is pH-dependent in the range of 3–10, indicating that the electrostatic interaction may be a controllable mechanism. To investigate the effect of pH value between 3 and 10, we also conducted one-way analysis of variance and *F*-test (more details can be found in electronic supplementary material, table S2). As illustrated in figure 5*b*, the adsorption capacity increased when pH < 7, but the adsorption capacity almost becomes stable under pH value between 7 and 8. The explanation of such phenomenon might be that TC exists as zwitterion at approximately 7, so the oxygen functional groups of G have little effect on TC. At pH > 8, the adsorption capacity of TC decreased. The increasing pH might facilitate the deprotonation of TC and G-KOH, suppressing the π–π interactions and cation–π bonding [32] between TC and G-KOH.

The adsorption capacities at various ionic strength range from 0 to 2.25 mmol l^−1^ was also analysed and compared. As shown in figure 5*c*, the adsorption capacity remained almost unchanged when the NaCl concentration changed from 0 to 2.25 mmol l^−1^. In order to describe the effect of ionic strength on adsorption capacity, we also used one-way analysis of variance and *F*-test to analyse the obtained data (more details can be found in electronic supplementary material, table S3). The result showed that at the significance level of 0.05 (confidence interval of 95%), there is no significant relationship between adsorption capacity and ionic strength over the range from 0 to 2.25 mmol l^−1^. The addition of NaCl almost had negligible influence over the adsorption capacity of TC, which might be due to the fact that the concentration of NaCl (0 ∼ 0.09 mg NaCl) is too low to have a notable influence. The present result is similar to that reported by Gao [53] in the NaCl concentration range of 20–100 mmol l^−1^. It is generally assumed that enough ionic strength would have a negative effect on the electrostatic interactions between the deprotonated carboxyl groups of GO and the positively charged amino group of tetracycline caused by the electrostatic screening effect [15].

## 9. Effect of coexisting ions

**Figure 6.**
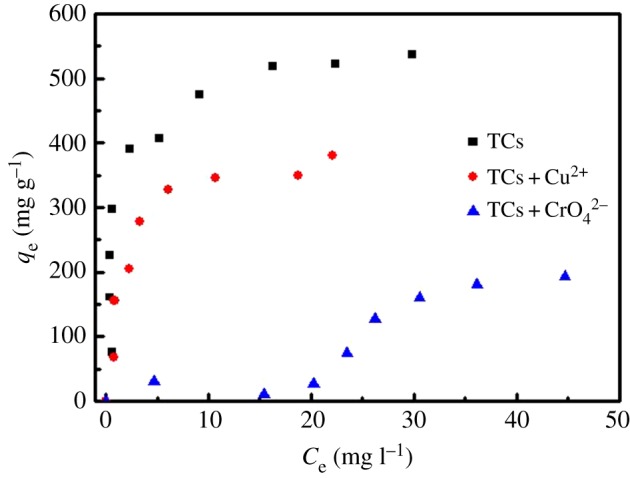
Equilibrium adsorption isotherms of coexisting Cu^2+^ and CrO_4_^2−^ with TC on G-KOH, respectively.

Under the practical circumstance, natural streams and waste effluents include many kinds of pollutants, especially toxic heavy metals. Accordingly, it becomes necessary to investigate the simultaneous adsorption behaviour and interaction during the co-adsorption process. Based on the above considerations, the Cu^2+^ and CrO_4_^2−^ was added into the tetracycline solution to study the effects of the coexisting metal ions and the preferential adsorption of G-KOH for different contaminants. The adsorption results of TC on G-KOH in the presence of Cu^2+^ and CrO_4_^2−^ is summarized in figure 6. The concentration of Cu^2+^ and CrO_4_^2−^ was kept constant as 20 mg l^−1^, during the entire experiment process. It was found that the presence of Cu^2+^ did not have any obvious effect on the adsorption performance of G-KOH at the beginning. But, with the increase of the concentration of TC, the adsorption capacity of TC on G-KOH decreased. The reduction might be caused by the competition for adsorption sites of G-KOH between TC and Cu^2+^ after the concentration of TC exceeded 40 mg l^−1^, which is consistent with the research by Ma *et al.* [54]. On the other hand, the presence of CrO_4_^2−^ has a negative effect on the adsorption performance in TC concentration range of 10–80 mg l^−1^, which may result from the interaction between the amino groups of TC and Cr(VI) as well as the interaction between carboxyl groups of G-KOH and Cr(VI). Chen *et al.* [53] found that the introduction of polyethyleneimine enhanced the adsorption capacity of Cr(VI), and they found that the removal of Cr(VI) depends on the electrostatic interaction between the Cr(VI), which are negatively charged, and the amine groups of the adsorbents, which are positively charged. Besides, in Ge & Ma's study [55], the absorbents have a higher selectivity for Cr(VI) than Cu(II). The adsorption performance of Chitosan (CS) for Cr(VI) is better than GO, which could be attributed to the active carboxyl groups. Hydroxy groups in GO for Cr(VI) adsorption are weaker than the interaction of amine groups in CS for Cr(VI) adsorption. Therefore, some Cr(VI) is reduced to Cr(III) by π electrons of G-KOH and then Cr(III) was adsorbed on the negatively charged groups (COO–) of G-KOH. Consequently, significant decrease of the adsorption capacity with the presence of CrO_4_^2−^ might be due to the interaction of TC and CrO_4_^2−^ as well as the interaction of GO and CrO_4_^2−^, which greatly reduced the adsorption capacity of G-KOH for TC.

## 10. Adsorption mechanism

π–π electron donor–acceptor interaction, electrostatic interaction and cation–π interaction are usually considered as the main driving force of adsorption between TC and graphene [56,57]. TC consists of four rings and hydrophilic groups, such as alcohol, phenol, ketone and amino. Graphene has delocalized conjugated π electrons and a lot of oxygen atoms in the form of hydroxyl, epoxy, carboxyl groups. The ring structure of TC and the surface of G-KOH could easily facilitate π–π stacking interaction. Additionally, led by the cation-induced polarization and electrostatic force, cation–π bonding takes place between the amino groups and graphene π-electron-rich area [15,58]. The adsorption capacity changed with the charges of TC when in cation condition and remained unchanged in zwitterion condition, implying that electrostatic interaction has a significant effect on adsorption performance of TC onto G-KOH. As shown in the FT-IR and XPS analysis results, new oxygen-containing functional groups are introduced into graphene sheets after activation treatment. As for the BET analysis results, the SSA of G-KOH increase greatly (from 138.2 to 512.65 m^2^ g^−1^), which could also provide more adsorption sites. The increase of adsorption sites on G-KOH facilitates π–π stacking interaction and cation–π interaction between G-KOH and TC, thereby improving the adsorption capacity to a large extent.

## 11. Conclusion

In this work, a novel graphene absorbent with excellent adsorption performance was synthesized by a simple KOH-activation method. Many micropores were introduced to graphene sheets in the activation process so that the SSA of G-KOH increased from 138.2 to 512.65 m^2^ g^−1^. The surface of G-KOH was also modified with large amounts of oxygen-containing functional groups, which improved the contact of G-KOH and TC and provided more adsorption sites. Higher SSA, hydrophilicity and more adsorption sites improved the adsorption capacity of TC on G-KOH to a great extent, which reached up to approximately 532.59 mg g^−1^. It is found that the Langmuir isotherm model was more suitable for the adsorption process and the adsorption kinetics was well represented by the PSO kinetic model. The effects of solid–liquid ratio, pH, ionic strength and coexisting ions were also investigated to facilitate understanding of the underlying adsorption mechanism. The results indicated that higher solid–liquid ratio and coexisting ions (Cu^2+^, CrO_4_^2−^) had obvious negative effects on adsorption performance, but the adsorption capacity of G-KOH is found insensitive to the change of pH and ionic strength. The adsorption mechanism was summarized as electrostatic interaction, π–π electron donor–acceptor interaction and cation–π interaction. Based on the analysis results, it can be concluded that KOH-activated graphene might be a promising adsorbent to remove TC in aqueous solutions.

## Supplementary Material

Supplementary Figures and Tables

## Data accessibility

The manuscript data were deposited at the Dryad Digital Repository: (http://dx.doi.org/10.5061/dryad.67b6q) [59].
