# Supplementary material for: Efficient removal of tetracycline with KOH-activated graphene from aqueous solution
Source: R Soc Open Sci. 2017 Nov 29;4(11):170731. doi: 10.1098/rsos.170731 (PMC5717638; doi:10.1098/rsos.170731)
Supplement: Supplementary Figures and Tables [file rsos170731supp1.docx]

**Supplementary document**

**Efficient removal of tetracycline with KOH-activated graphene from aqueous solution**

**Journal:**

Royal Society Open Science

**Manuscript Title:**

Efficient removal of tetracycline with KOH-activated graphene from aqueous solution

**Authors’ names:**

Jie Ma^1^, Yiran Sun^1^, Fei Yu^1,2*^

1 State Key Laboratory of Pollution Control and Resource Reuse, College of Environmental Science and Engineering, Tongji University, 1239 Siping Road, Shanghai 200092, P. R. China. Tel: 86-21-6598 1831; E-mail: [jma@tongji.edu.cn](mailto:jma@tongji.edu.cn)

2 College of Chemistry and Environmental Engineering, Shanghai Institute of Technology, Shanghai 2001418, China; E-mail: [fyu@vip.163.com](mailto:fyu@vip.163.com)

**Corresponding author:**

Fei Yu^1,2*^

College of Chemistry and Environmental Engineering, Shanghai Institute of Technology, Shanghai 2001418, China;

E-mail: [fyu@vip.163.com](mailto:fyu@vip.163.com)

Fig. S1. XPS survey scans of G and G-KOH

Fig. S2. FTIR spectra of G and G-KOH

Fig. S3. Raman spectra of G and G-KOH

Fig. S4. N_2_ adsorption/desorption isotherms of G and G-KOH.

Table S1. The relationship between adsorption process and R_L_

| Value of R_L_ | adsorption process |
| --- | --- |
| R_L_ >1 | Unfavorable |
| R_L_ =1 | Linear |
| 0< R_L_ <1 | Favorable |
| R_L_ = 0 | Irreversible |

Table S2. One-way analysis of variance for pH

| Source of variance | SS | df | MS | F-value |
| --- | --- | --- | --- | --- |
| pH | 3434.13 | 7 | 490.59 | 0.85 |
| error | 4618.27 | 8 | 577.28 |  |
| total | 8052.40 |  |  |  |

Table S3. One-way analysis of variance for ionic strength

| Source of variance | SS | df | MS | F-value |
| --- | --- | --- | --- | --- |
| ionic strength | 385.83 | 7 | 55.12 | 0.14 |
| error | 3052.94 | 8 | 381.62 |  |
| total | 3438.77 |  |  |  |

Note:

SS: sum of square of deviation;

df: degree of freedom;

MS: mean square

Table S4 Adsorption kinetics model parameters for TC of G and G-KOH

| Adsorbent | qe,exp  (mg/g) | PFO model | | | PSO model | | | Intra-particle diffusion model | | |
| --- | --- | --- | --- | --- | --- | --- | --- | --- | --- | --- |
|  |  | k_1_  (/min) | qe,cal  (mg/g) | R^2^ | k_2_  (/min) | qe,cal  (mg/g) | R^2^ | K_i_  (g/mg/min^0.5^) | C  (mg/g) | R^2^ |
| G | 231.39 | 0.0051 | 76.71 | 0.36 | 0.0003 | 250.00 | 0.99 | 3.75 | 124.77 | 0.52 |
| G-KOH | 537.60 | 0.0035 | 179.60 | 0.75 | 0.0001 | 500.00 | 0.98 | 5.36 | 333.29 | 0.79 |

Table S5 Cost of reagents for manufacturing G-KOH

| Adsorbent | Cost(/kg)^a^ |
| --- | --- |
| KOH | 40.0 |
| Graphite Oxide | 1000.0 |
| HCl | 11.8 |

^a^ Prices from Sinopharm Chemical Reagent Co., Ltd. (Shanghai, China)

Table S6 Cost of different adsorbents

| Adsorbent | Cost(¥/kg) ^a^ |
| --- | --- |
| Standard commercial activated cabons | 61.0 |
| G | 1020.0 |
| G-KOH | 1976.9 |

^a^ Prices from Sinopharm Chemical Reagent Co., Ltd. (Shanghai, China)
